# Human prolyl hydroxylase expression in uterine leiomyoma during the menstrual cycle

**DOI:** 10.1186/1477-7827-10-111

**Published:** 2012-12-17

**Authors:** Masaaki Iwahashi, Yasuteru Muragaki, Kazuhiko Ino

**Affiliations:** 1 Department of Obstetrics and Gynecology, Wakayama Medical University, Wakayama, 641-0012, Japan; 2 Department of Pathology, Wakayama Medical University, Wakayama, 641-0012, Japan

**Keywords:** Prolyl hydroxylase, Human uterine myometrium, Leiomyoma, Immunohistochemistry

## Abstract

**Background:**

To investigate the role of prolyl hydroxylase (PH), a key enzyme of collagen synthesis, in human uterine leiomyoma, PH expression was determined in the normal uterine myometrium and the leiomyoma tissues during the menstrual cycle.

**Methods:**

The tissues were obtained from 40 regularly cycling women (aged 29 to 53 yr) who were undergoing abdominal hysterectomy for symptomatic uterine leiomyoma. Immunohistochemistry for human PH with specific monoclonal antibody was used for analysis.

**Results:**

Immunohistochemical staining for PH revealed intense staining of leiomyoma cells in the uterine leiomyoma throughout the menstrual cycle, as compared with the adjacent normal myometrium. In the secretory phase, weak or no immunostaining for PH was detected in the normal myometrial tissues.

**Conclusions:**

These results suggest that increased expression of PH might play an role in the physiology of uterine leiomyoma during the menstrual cycle.

## Background

The extracellular matrix (ECM) is considered to play an important role in the stability of tissues and in regulating the growth and differentiation of cells [1,2].

Synthesis, accumulation, and catabolism of the ECM occur during wound healing and during the initiation and progression of numerous diseases [3]. Moreover, it is generally acknowledged that the ECM does not function as a mere passive scaffold for connective tissue within organ architecture. It is also suggested that ECM plays an ‘informational’ role through a network of interactions between cells and signal molecules that is of primary importance in the control of cellular proliferation and motility during histogenesis, for the maintenance of tissue homeostasis and in cancer development. The ECM of uterine leiomyoma has been studied [4-7], but changes of the collagen metabolism in uterine tissues are not fully understood. Therefore, the precise control of ECM metabolism in the uterine myoma and myometrium is critical for understanding the pathophysiology and development of uterine leiomyoma.

Most of the 4-hydroxyproline in mammalian proteins is found in the -X-4Hyp-Gly- sequences of the 29 currently known collagen types and more than 20 additional proteins with collagen–like triple-helical domains [8,9]. 4-Hydroxyproline residues have a vital role in providing the collagen triple helices with thermal stability. Non-hydroxylated collagen polypeptide chains cannot form functional molecules *in vitro*, and almost complete hydroxylation of the proline residues in –X-Pro-Gly- triplets is required for the generation of a molecule that is stable at human body temperature. This hydroxylation is catalyzed by collagen prolyl hydroxylase (PH) located within the lumen of the endoplasmic reticulum [10]. Therefore, in the present study, we investigated the distribution of human PH, a key enzyme in the synthesis of collagen [11,12], in human uterine leiomyoma by immunofluorescent staining.

## Methods

This project was approved by the Committee on Investigations Involving Human Subjects of Wakayama Medical University. Informed consent was obtained from each subject after the purpose and nature of the study had been fully explained.

### Tissues

Leiomyomas and matched myometrium were processed for immunohistochemistry. The tissues were obtained from 40 premenopausal regularly cycling women (aged 29 to 53 yr) who were undergoing abdominal hysterectomy for symptomatic uterine leiomyoma. None of the patients received any hormone therapy before operation. The stage of menstrual cycle was determined by histological dating of the endometrium for all secretory phase samples. Proliferative phase samples were dated by either dating of the endometrium or date of last menstrual period. The leiomyoma and corresponding myometrium specimens from the proliferative (*n* = 20) and secretory (*n* = 20) phase were studied. No submucosal leiomyomas were collected so as to avoid possible contamination with endometrium. Three distant samples were collected from each leiomyoma and adjacent normal myomerial tissues. These tissues were immediately frozen in liquid nitrogen.

### Primary antibodies

We used the monoclonal antibodies (mAbs) against of hPH (anti-hPH(β); horseradish peroxidase-conjugated Fab’ (Fab’-HRP)), which recognized the β subunit of prolyl hydroxylase, were prepared as described by Bai et al. [13]. Preparation of the antibodies has been performed as described previously [14]. The specificity of the antibodies was determined by immunoblotting or by inhibition in an enzyme-linked immunosorbent assay.

### Immunohistochemistry

Immunohistochemical analysis was performed by the standard indirect immunofluorescence method. In brief, 3-μm frozen sections were rehydrated in phosphate-buffered saline (PBS) at room temperature and then incubated with the primary antibody (diluted 1: 100 in PBS) for 12 h at 4°C in a humidified chamber. After incubation, the sections were washed twice in PBS for 3 min. Each section was then incubated for 1 h at room temperature with human plasma-preabsorbed, fluorescein isothiocyanate-conjugated goat antibodies against mouse immunoglobulins diluted 1:100 in PBS (Organon Teknik, Co., West Chester, PA).

**Figure 1:**
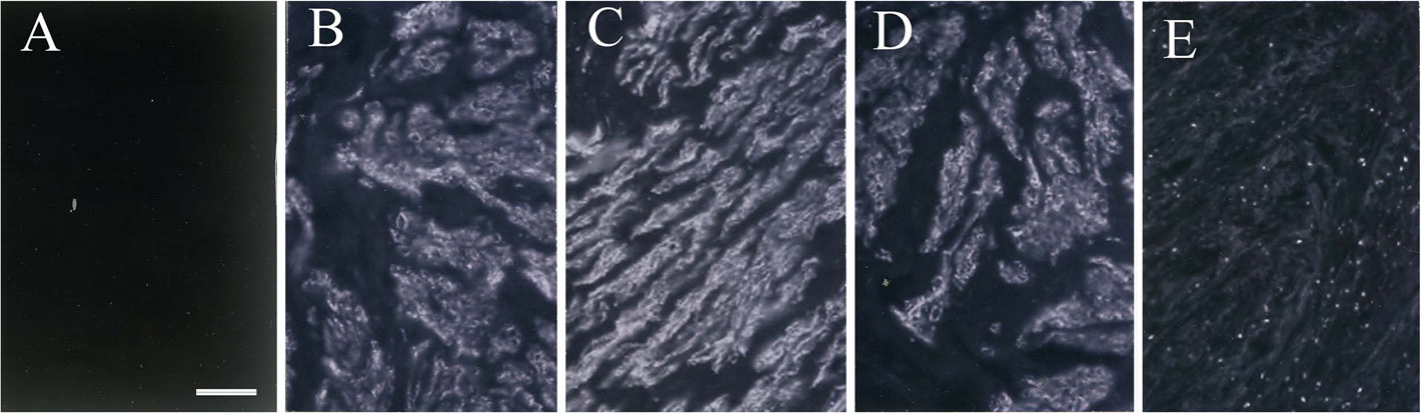
**Immunofluorescence micrographs of human uterine leiomyoma and myometrium with immunostaining by mAbs specific for human PH.** The micrographs of leiomyoma are **B** and **D**, and the micrographs of myometrium are **C** and **E** during the menstrual cycle (proliferative phase: **B** and **C**; secretory phase: **D** and **E**). No immunofluorescence was recognized in the control section (**A**). (Original magnification, X 125) Scale bar represents 50 μm.

Control sections were stained with goat antibodies against mouse immunoglobulin G without prior application of the primary antibodies. No immunofluorescence was recognized in control sections (Figure 1A). When the mAb was first allowed to react with an excess of hPH , no immunostaining was observed (data not shown).

## Results

Immunostaining with the mAbs against hPH showed intense granular pattern in the cytoplasm of leiomyoma cells as compared with the normal uterine myometrial tissues throughout the menstrual cycle consistently (Figure 1B and D). Immunostaining for hPH showed intense granular pattern in the cytoplasm of the normal myometrial cells in the proliferative phase (Figure 1C). However, weak or no immunostaining for hPH was detected in the normal myometrial tissues in the secretory phase (Figure 1E).

The intensity of staining of the uterine tissues from the menstrual cycle by the antibodies against hPH was subjectively graded from 1+ to 3+ and the results are summarized in Table 1.

**Table 1:** Immunostaining with antibodies for human prolyl hydroxylase in the human uterine leiomyoma and myometrium

	**Phase**	
	**Proliferative (*n*** **= 10)**	**Secretory (*n*** **= 10)**
Leiomyoma	+++	++
Myometrium	++	+/−

Intensities of immunostaining were int graded subjectively.

−, no staining; +, weak staining; ++, intense staining; +++, very intense staining.

## Discussion

In the present study, we investigated the immunolocalization of hPH in the human uterine tissues obtained during the menstrual cycle. Immunostaining for hPH was clearly apparent in the cytoplasm of the uterine leiomyoma cells and the normal myometrial cells during the menstrual cycle. Especially, in the uterine leiomyoma, the strong expression of hPH was detected, as compared with the normal adjacent myometrium during the menstrual cycle. These findings suggest that leiomyoma cells might synthesize collagens more actively than the normal myometrial cells throughout the menstrual cycle consistently.

We have previously suggested that the increased expression of the various collagens, such as type III, IV and V collagens, is recognized by immunohistochemistry and sodium dodecyl sulfate-polyacrylamide gel electrophoresis in the human uterine leiomyoma, as compared with the normal uterine myometrium during the menstrual cycle [15,16]. Therefore, increased these collagens expression might play an important role in the morphologic and functional characteristics of human uterine leiomyoma.

PH, a key enzyme in the hydroxylation of proline to hydroxyproline during the synthesis of collagen [11,12], is a tetramer of two α and β subunits [17]. A cDNA sequence for the β subunit of hPH has been found to be highly homologous with that for a rat protein disulfide isomerase, which is regarded as the catalyst *in vivo* for formation of disulfide bonds in the biosynthesis of various secretory proteins [17,18]. Therefore, immunoreactivity of the β subunit may reflect the enzymatic activity of PH in collagen metabolism.

Recently, it has been suggested that oxygen-dependent hydroxylation of hypoxia-inducible factor (HIF)- α subunits by PH domain (PHD) proteins signals their polyubiquitination and proteasomal degradation, and a critical role in regulating HIF abundance and oxygen homeostasis. While oxygen concentration plays a major role in determining the efficiency of PHD-catalyzed hydroxylation reactions, many other environmental and intracellular factors also significantly modulate PHD activities. In addition, PHDs may also employ hydroxylase-independent mechanisms to modify HIF activity. Functionally, different PHD isoforms may differentially contribute to specific pathophysiological processes, including angiogenesis, erythropoiesis, tumorigenesis, diabetes, and cell growth, differentiation and survival [10,19-25]. Therefore, our findings support the hypothesis that HIF-PH systems might be involved in the pathogenesis of uterine leiomyoma during the menstrual cycle [26,27].

## Conclusions

An increased hPH expression was found in the uterine leiomyoma cells of the uterine fibroids throughout the menstrual cycle. Thus, these consistent expression of hPH might play a role in the physiology of human uterine fibroids.

This study provides some clues to understanding the pathogenesis of human uterine leiomyoma in terms of the, ECM metabolism. Further work is needed to elucidate the mechanisms regulating the HIF-PH expression in the human uterus during the menstrual cycle and the medical treatments.

## Abbreviations

ECM: Extracellular matrix; HIF: Hypoxia-inducible factor; Mabs: Monoclonal antibodies; PBS: Phosphate-buffered saline; PH: Prolyl hydroxylase.

## Competing interest

The authors have no competing interest to declare.

## Authors’ contributions

MI carried out the Immunohistochemical studies, and participated in the design and drafted the manuscript. YM carried out the production of the monoclonal antibodies and evaluated the result of immunohistochemical staining. KI participated in the design of the study, and conceived of the study, and participated in its design and consideration and drafted the manuscript. All authors have read and approved the final manuscript.

## Authors’ information

MI is the assistant professor of the Obstetrics and Gynecology in Wakayama Medical University. YM is the professor of the Pathology in Wakayama Medical University. KI is the professor of the Obstetrics and Gynecology in Wakayama Medical University.
